# Sustained and Cost Effective Silver Substrate for Surface Enhanced Raman Spectroscopy Based Biosensing

**DOI:** 10.1038/s41598-017-07186-9

**Published:** 2017-07-31

**Authors:** Jian Ju, Wei Liu, Clint Michael Perlaki, Keren Chen, Chunhua Feng, Quan Liu

**Affiliations:** 1 School of Chemical and Biomedical Engineering, Nanyang Technological University, 70 Nanyang Drive, Singapore, 637457 Singapore; 2 School of Environment and Energy, South China University of Technology, Guangzhou, 510006 China

## Abstract

While surface enhanced Raman spectroscopy (SERS) based biosensing has demonstrated great potential for point-of-care diagnostics in the laboratory, its application in the field is limited by the short life time of commonly used silver based SERS active substrates. In this work, we report our attempt towards SERS based field biosensing, involving the development of a novel sustained and cost-effective substrate composed of silver nanoparticles protected by small nitrogen-doped Graphene Quantum Dots, i.e. Ag NP@N-GQD, and its systematic evaluation for glucose sensing. The new substrate demonstrated significantly stronger Raman enhancement compared to pure silver nanoparticles. More importantly, the new substrate preserved SERS performance in a normal indoor environment for at least 30 days in both the wet and dry states, in contrast to only 10 days for pure silver nanoparticles. The Ag NP@N-GQD thin film in the dry state was then successfully applied as a SERS substrate for glucose detection in mouse blood samples. The new substrate was synthesized under mild experimental conditions, and the cost increase due to N-GQD was negligible. These results suggest that the Ag NP@N-GQD is a cost-effective and sustained SERS substrate, the development of which represents an important step towards SERS based field biosensing.

## Introduction

Surface enhanced Raman scattering (SERS) is an ultra-sensitive spectroscopic technique^1, 2^, which has been used for sensing in a wide range of biomedical fields, such as toxicology^3^, microbiology^4^, and disease prognosis and diagnosis^5, 6^. Because of requiring only a small sample volume and high sensitivity and selectivity, SERS based techniques have shown great potential as a point-of-care platform for point-of-care diagnostics of diseases in field tests^7, 8^. However, one major drawback of conventional silver based SERS substrates is their poor physical stability and strict storage conditions for SERS activity preservation, which has limited the application of SERS based field biosensing^9, 10^.

It is well known that the SERS phenomenon depends on strong localized surface plasmon resonance (LSPR) at the surface of metallic nanostructures such as silver and gold^11–16^. While gold based SERS substrates are more stable, the cost is much higher and the SERS performance is typically worse than that of silver based counterparts^17^. Up to now, a variety of silver nanostructures with different shapes, such as spheres^18^, rods^19^, sunflower-like^20^ and cubes^21^, have been successfully synthesized and usually exhibit high SERS performance. However, one major drawback of silver based SERS active nanostructures is their poor physical stability. An oxidized layer will be formed in a short time once pure silver is exposed to air, which makes sustained SERS performance in the long term difficult. Conventionally, in order to improve the performance and stability of SERS based sensors^9, 10^, metallic nanostructures are usually covered by a protective agent such as metal oxide (Fe_3_O_4_, ZnO)^13, 22^, semiconductor quantum dots (CdSe, CdS) or carbon materials^23, 24^. Among these materials, graphene often exihibits a highter SERS effect after target molecules are adsorbed on a monolayer graphene surface^25–28^. This SERS enhancement is also explained as a type of chemical enhancement^29^, and the key factor influencing the SERS efficiency is the number of graphene layers on the surface of the metal NP due to the “first layer effect” of the graphene^30, 31^. Recently, small-size graphene quantum dots (GQD) that were fabricated from large graphene sheets have attracted much attention due to their low biological toxicity, good biocompatibility and good surface grafting ability^32–37^. Small-size zero-dimensional (0D) GQD are able to wrap around the surface of metal nanoparticles by electrostatic bonding or π−π cooperative interactions. Thus, such metal NP wrapped by GQD could work as an effective substrate for SERS measurements. In addition, it has been shown that carbon materials can delay the oxidation of metal nanoparticles in common storage environment^35, 38^. Therefore, such zero-dimensional (0D) GQD materials could be used to improve and preserve the performance of Ag based SERS substrates in the longer term. However, the capability of GQD or related materials preserving the SERS performance of silver nanoparticles has not been quantitatively evaluated, and the optimal configuration of such composite substrates has not been reported in the literature to our knowledge.

In this article, we report the development of a novel Ag based SERS substrate consisting of nearly uniformly distributed Ag nanoparticles (Ag NPs) protected by small-size nitrogen-doped GQD (N-GQD) and its characterization of long-term SERS performance for up to 30 days for the first time. N atom doped GQD carries more negative charge than GQD, thus it can donate its lone pair of electrons to metal nanoparticles, which would increase the binding energy between N-GQD and metallic nanoparticles surfaces^35, 38, 39^. As a result, the Ag NPs are protected by N-GQD in the form of small sheets in the outer layer, which can dramatically improve the surface stability of the composite nanoparticles and prevent Ag nanoparticles from aggregating even in the dry state. It is demonstrated in this work that the new Ag NP@N-GQD can be used as a stable SERS substrate to sensitively detect not only Rhodamine 6G (R6G) in the wet state and glucose in the dry state, but also glucose in mouse blood samples. The optimal configuration of Ag NP@N-GQD was experimentally determined. The results of the time-profile study show that Ag NP@N-GQD in both the wet and dry states can maintain 68% and 50% of their initial SERS intensity in normal indoor conditions for at least 30 days, respectively. The new substrate was synthesized under mild experimental conditions and the cost increase compared to pure Ag NPs was negligible. Such a sustained and cost effective SERS substrate represents an important step towards SERS based field biosensing.

## Results and Discussion

### Characterization of Ag NPs and Ag NP@N-GQD

**Figure 1:**
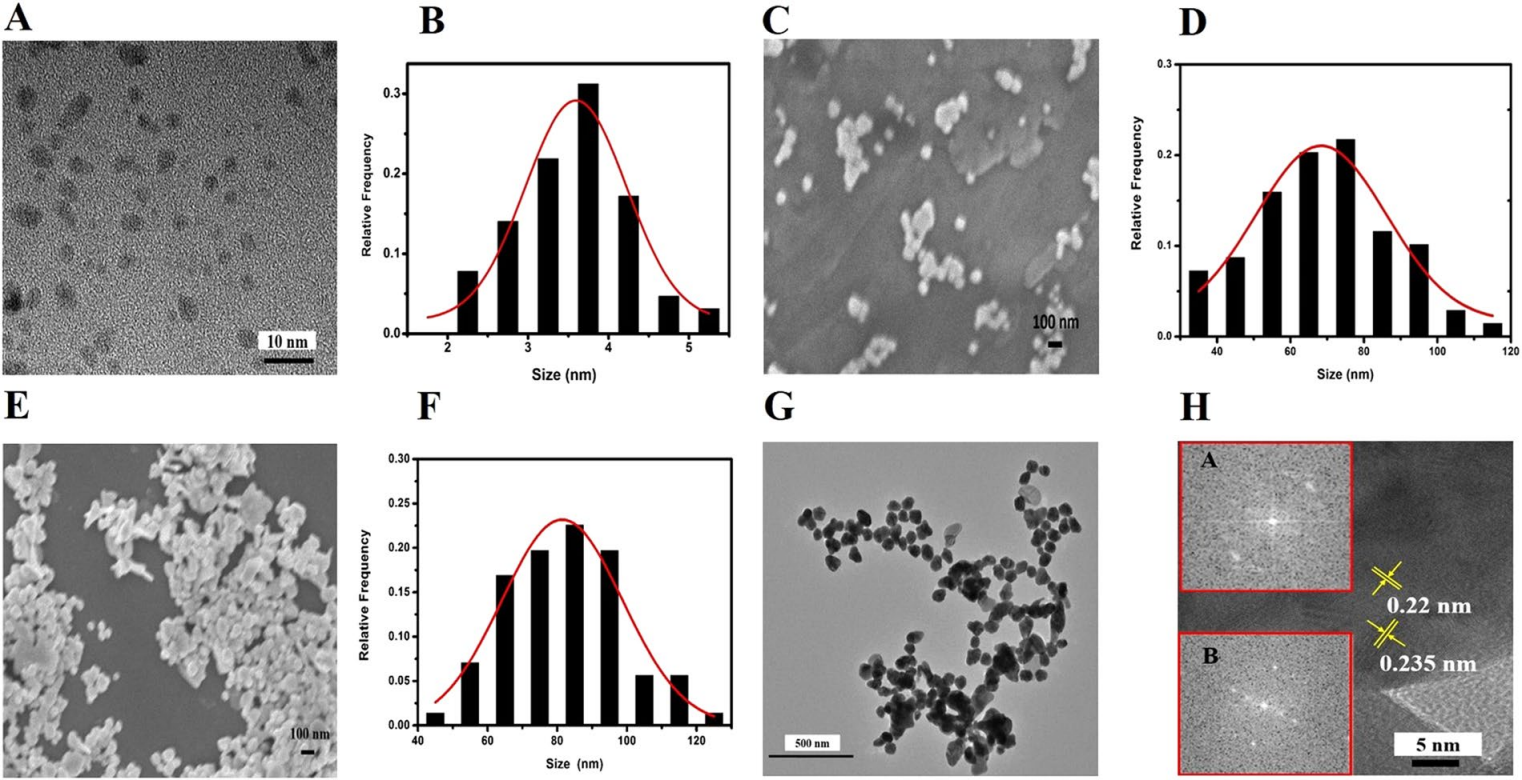
(**A**) TEM images of N-GQD, and the SEM images of the synthesized (**C**) Ag NPs and (**E**) Ag NP@N-GQD. The images (**B**,**D** and **F**) show the size histograms of N-GQDs, Ag NPs and Ag NP@N-GQD derived from SEM images, respectively. (**G**) TEM images of the as-synthesized Ag NP@N-GQD. (**H**) HRTEM images of Ag NP@N-GQD and FFT patterns of N-GQDs and N-GQDs Ag NP shown in inset a and b, respectively.

The morphologies of the synthesized N-GQD, Ag NPs and Ag NP@N-GQD were characterized by TEM and SEM as shown in Fig. 1. The TEM image of the N-GQD, Fig. 1(A), reveals they formed small sheets with a narrow size distribution. The SEM images of the Ag NPs in the dry state, as in Fig. 1(C), shows various shapes of nanoparticles mixed together due to the weak morphology control performance of the surfactant TX-100. The SEM image of Ag NP@N-GQD in Fig. 1(E) clearly illustrates that the Ag NP@N-GQD in the dry state shows a slightly narrower particle size distribution and after N-GQD is coated on the Ag NP surface. The size histograms shown in Fig. 1(B, D and F) suggest that the average diameters of the N-GQD, Ag NPs and Ag NP@N-GQD are about 3.8 nm, 75 nm and 85 nm, respectively, as evaluated by analyzing the images of 100 individual particles for each type of NP. Moreover, from the TEM image of Ag NP@N-GQD shown in Fig. 1(G), we find the trend of NP aggregation increases with the addition of N-GQDs due to the electrostatic bonding or π−π cooperative interactions between metal NPs. Figure 1(H) shows the HRTEM image and fast Fourier transform (FFT) pattern of Ag NP@N-GQD. The HRTEM image reveals the crystalline organization of both N-GQDs and the *fcc* structure of Ag NPs. The lattice spacing of 0.22 nm and 0.235 nm were measured from FFT (inset a and b), which correspond to the (1 1 $\bar{2}$ 0) lattice fringes of graphene^36^ and (1 1 1) planes of the Ag NP structure^40^, respectively. These values were calculated using a commercial software (Digital Micrograph, Gatan, INC, Pleasanton, California, USA). This indicates that N-GQDs had successfully wrapped around the surface of silver NPs.

**Figure 2:**
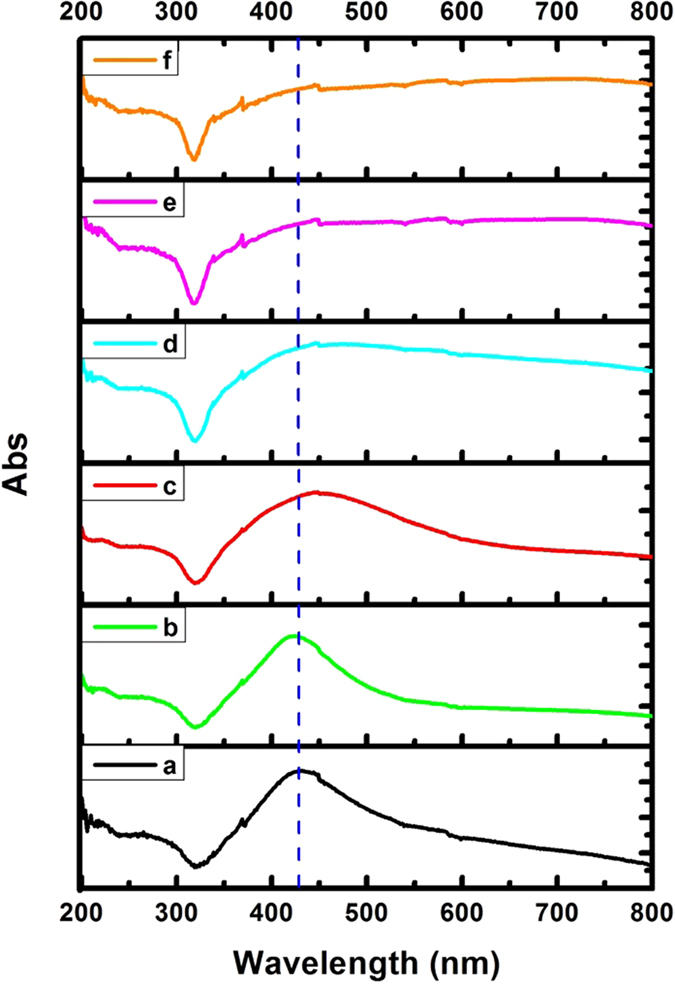
UV-Visible absorption spectra of the synthesized Ag NPs (curve a) and Ag NP@N-GQD (curves b–f) with various mass ratios (Q_N-GQD_/Q_AgNO3_ = 0, 0.04, 0.08, 0.12, 0.16 and 0.2, respectively).

Different volumes (0, 2, 4, 6, 8 and 10 mL) of N-GQD (0.2 mg mL^−1^) was added into the reaction solution that contained 10 mg AgNO_3_ to achieve a range of mass ratios (Q_N-GQD_/Q_AgNO3_ = 0, 0.04, 0.08, 0.12, 0.16 and 0.2, respectively) and the samples were labeled a through f, respectively. The UV–visible absorption spectra of the aqueous dispersion of Ag NPs (curve a) and Ag NP@N-GQD (curves b-f) are shown in Fig. 2. It can be seen that the absorption spectra of pure Ag NPs (curve a) exhibits a typical peak at 432 nm as indicated by the dashed vertical line. However, the Ag NP@N-GQD (curves b–f) shows an absorption peak slightly red shifted. For example, the peak wavelength changed to 449 nm in curve c. This red shift indicates the aggregation of Ag NPs after decoration with N-GQD, which is consistent with the observation in the SEM and TEM images.

**Figure 3:**
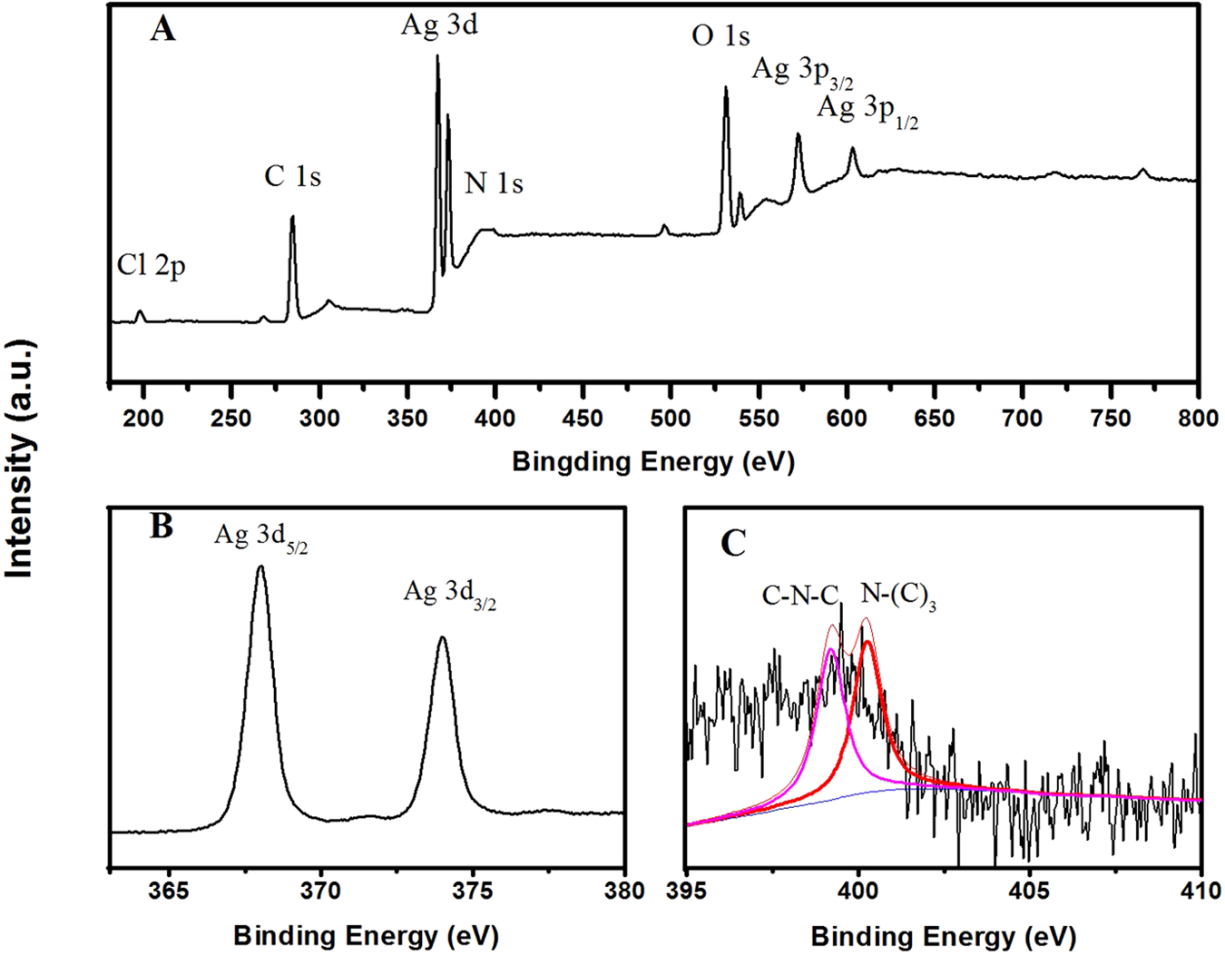
XPS spectra of (**A**) the synthesized Ag NP@N-GQD, (**B**) Ag 3d and (**C**) N 1s.

Figure 3 shows the X-ray photoelectron spectrum (XPS) of Ag NP@N-GQD using C 1s as the reference. The XPS spectrum in Fig. 3(A) clearly shows the binding energy peaks from Ag 3d, C 1s, N 1s and O 1s, indicating the presence of Ag, C, N, O elements in the composition of Ag NP@N-GQD^35, 41^. From Fig. 3(B), which shows the high resolution XPS of Ag 3d, it can be seen there are two major peaks at 367.6 and 373.6 eV, which can be ascribed to the binding energies of Ag 3d5/2 and Ag 3d3/2, respectively. This result indicates the strong presence of silver in the metallic state^41^. The XPS spectrum of N 1s presented in Fig. 3(C) that can be fit to the summation of contributions by two different components at 399.2 and 400.5 eV are assigned to C−N−C and N−(C)_3_ bonds, respectively^35, 42^.

### Characterization of the SERS performance of Ag NPs and Ag NP@N-GQD

The optimal experimental conditions were found by the results of UV and SERS. As shown in Fig. 2, the UV–vis spectra indicates the shape change and size distribution. It can found that the absorption peak of the Ag NP@N-GQD (samples b through f) have an obvious red shift compared with pure Ag NPs (sample a). This result indicates that the size of composite nanoparticles has increased due to the varying amount of N-GQD decorated on the surface of the Ag NPs. When the mass ratio (Q_N-GQD_/Q_AgNO3_) is greater than 0.08, the absorption peak of Ag NP@N-GQD (samples d through f) become wider and flatter^43, 44^. This may be attributed to the fact that more N-GQD covering the surface of the Ag NPs result in the aggregation of nanoparticles^32, 35^.

**Figure 4:**
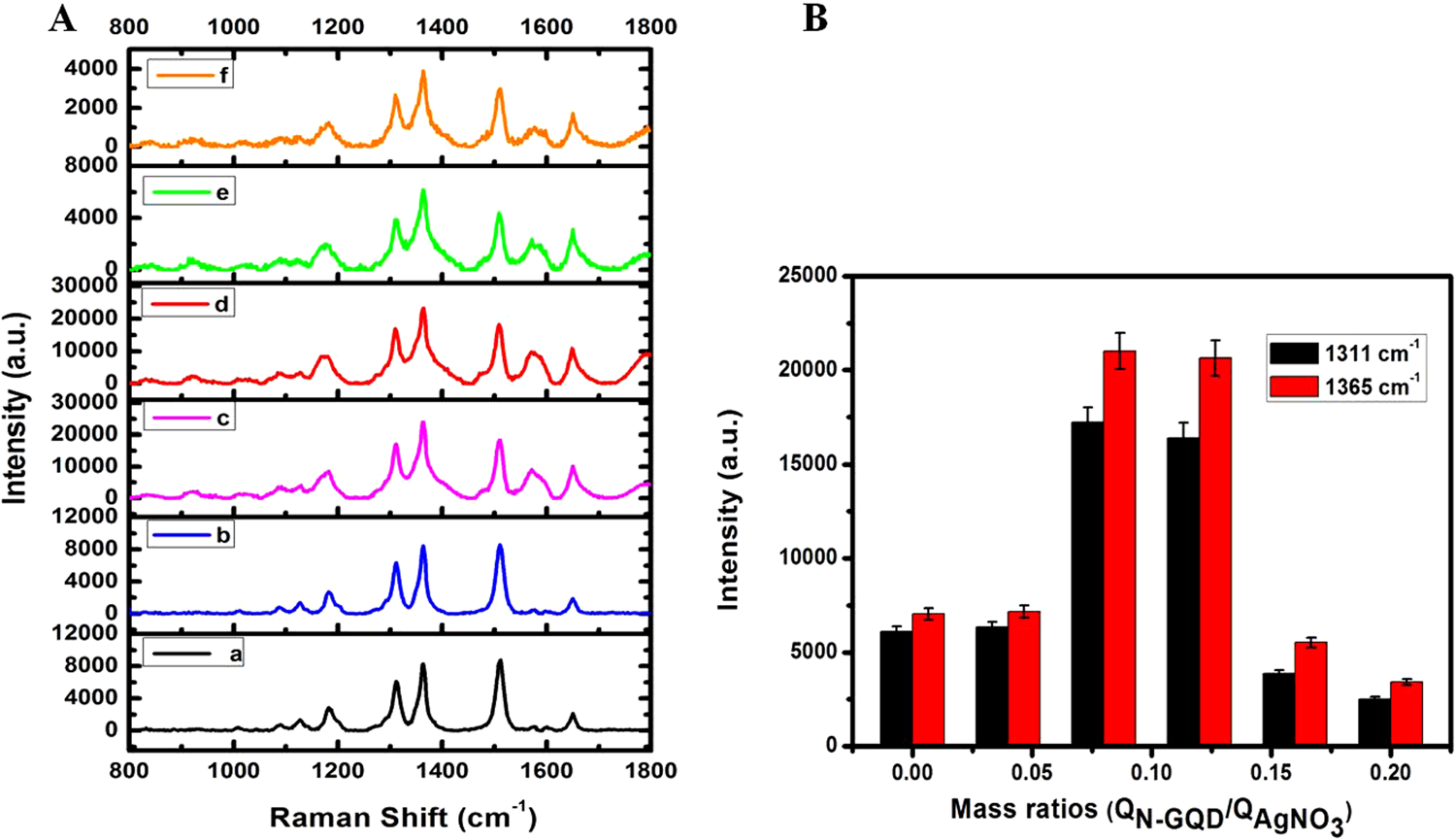
SERS spectra of R6G (1 μM) adsorbed on freshly prepared Ag NPs (curve a) and Ag NP@N-GQD (curves b–f) with various mass ratios (Q_N-GQD_/Q_AgNO3_ = 0, 0.04, 0.08, 0.12, 0.16 and 0.20, respectively, from a to f) excited by laser at 785 nm. (**B**) The histograms were generated from the Raman intensity valuesat 1311 and 1365 cm^−1^. The magnitude of the error bar is equal to the standard deviation for each mass ratio.

Figure 4 shows the measured SERS spectra of aqueous R6G (10^−6^ M) solution adsorbed on the synthesized Ag NPs (curve a) and Ag NP@N-GQD (curves b through f). The G peak of the monolayer graphene at 1588 cm^−1^ can be observed with the addition of N-GQD at different concentrations (from curves a through f)^45^. In order to achieve a fair comparison, two types of nanoparticles with nearly identical concentrations (1 mg mL^−1^) were used, and all the experiments were conducted under the same conditions. To investigate the SERS performance of Ag NPs and Ag NP@N-GQD, 100 μL of each type of nanoparticle (with a concentration of 1 mg mL^−1^) was mixed with 100 μL aqueous R6G (10^−6 ^M) solution in a 2-mL centrifuge tube. Most prominent Raman peaks can be observed in all samples, such as those at 615 cm^−1^, 775 cm^−1^, 1185 cm^−1^, 1311/1365 cm^−1^ and 1508 cm^−1^, which can be assigned to C─C─C ring in-plane bending, CH out-of-plane bending, C─O─C retching, C─C∕C─N stretching and aromatic C─C stretching, respectively^46, 47^. It can be seen that the SERS performance of Ag NP@N-GQD is comparable to that of Ag NPs when the mass ratio is 0.04 (curve b) and the enhancement factor increases with the mass ratio until reaching the maximum at a mass ratio of 0.08 (curve c). Surprisingly, Ag NP@N-GQD show even better SERS performance than pure Ag NPs when the mass ratio is ranged from 0.04 (curve b) to 0.12 (curve d). According to the previous reports, chemical enhancement and electromagnetic enhancement are the two well known mechanisms behind SERS. Graphene plays a key role for studying the chemical enhancement mechanism due to the functionalized chemical bonds that can promote light-induced charge transfer between adsorbed probe molecules and the SERS substrate^28, 29, 48^. N-GQDs, which are essentially small pieces extracted from the graphene matrix, induce SERS enhancement very likely by the same mechanism, i.e. the chemical enhancement caused by the electron transfer between N-GQD and and R6G molecules adsorbed on the surface of Ag NPs^29, 49, 50^. This explanation can be also confirmed by the observation that the SERS performance of Ag NP@N-GQD for enhancing the main peak of R6G is about three times that of pure Ag NPs in this study. Such a small enhancement factor can only be explained by chemical enhancement since the electromagnetic enhancement mechanism typically yields an enhancement factor of several orders of magnitude. The aromatic stretching vibration at 1650 cm^−1^ is attributed to R6G molecules stably adsorbed on the Ag NP’s surface^45^. However, once the mass ratio (Q_N-GQD_/Q_AgNO3_) is greater than 0.12 (curves e and f), the SERS performance starts to drop. This phenomenon could be attributed to the likely thicker N-GQD layer with a larger mass ratio forming a sealed N-GQD shell that prevented direct contact of R6G molecules with the Ag NP surface^47, 51^. Thus, the mass ratio Q_N-GQD_/Q_AgNO3_ = 0.08 was used as the optimal value.

We also calculated the analytical enhancement factor (*EF*) of AgNP and AgNP@N-GQD (Q_N-GQD_/Q_AgNO3_ = 0.08) for R6G molecule^46^. We estimated that the *EF* of AgNP and AgNP@N-GQD SERS signals relative to the ordinary Raman measurement (*EF*_SERS∕Raman; R6G_) are about 7.05 × 10^5^ and 2.06 × 10^6^, respectively, which is close to those reported in literature^46^.

### SERS detection of glucose using Ag NP@N-GQD

**Figure 5:**
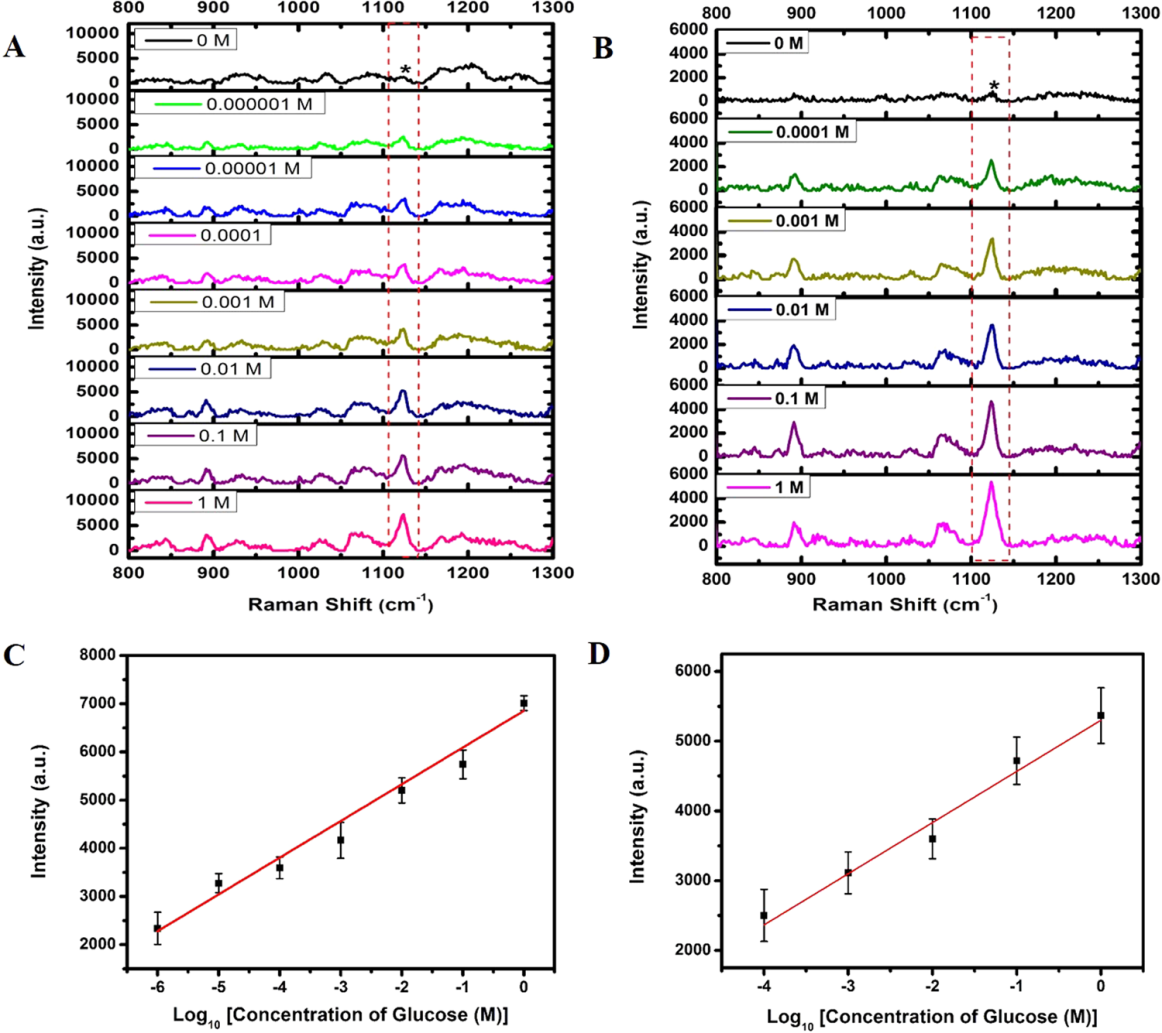
Representative SERS spectra of the aqueous glucose solution mixed with dried 1-DT-modified (**A**) Ag NP@N-GQD (freshly made) and (**B**) Ag NPs (freshly made) on the surface of a clean aluminum foil, respectively. The excitation wavelength was 785 nm with a power density of 9.09 W/mm^2^ and an exposure time of 5000 ms. SERS intensity for the peak at 1123 cm^−1^ of the (**C**) Ag NP@N-GQD and (**D**) Ag NPs dependence on the concentration of glucose with the range of (10^−6^ to 1 M) and (10^−4^ to 1 M), respectively. Each data point represents the average value from five SERS spectra measured from different locations. Error bars indicate the standard deviations.

Figure 5 demonstrates the SERS spectra measured from glucose solutions at a range of concentrations. Figure 5(A) clearly shows that the Raman peaks at 889, 1073 and 1125 cm^−1^ are attributed to the 1-DT layer coated on the surface of Ag NP@N-GQD and the Raman peaks of the characteristic glucose are 1076 cm^−1^ (C-C stretching) and 1123 cm^−1^ (C-O-H deformation)^52^. The SERS peak at 1123 cm^−1^ was utilized for the assessment of glucose concentration. The calibration curve, which is the SERS peak intensity as a function of glucose concentration for a range of 10^−6^ to 1 M, is shown in Fig. 5(C), in which every sample was measured five times each at a different location. The standard regression equation is determined by a commercial software (Origin 8.0, OriginLab, Northampton, MA, USA), which was found to be ${{\boldsymbol{I}}}_{(1123{{\rm{cm}}}^{-1})}$ = 762.4 × ***C***_*glucose*_ + 6854.8 (*R* = 0.979). The detection limits of glucose with this sensing system are estimated to be 0.1 μM using the IUPAC method and 0.022 μM using the AOAC method, respectively, which is lower than several earlier reported Ag nanomaterials and even gold based SERS sensors as summarized in Table 1. The SERS performance of pure Ag NPs for glucose detection was also studied. Figure 5(B) shows the SERS spectra measured from glucose solutions at a range of concentrations, and a linear correlation was obtained over a concentration range from 10^−4^ to 1 M as shown in Fig. 5(D). The standard regression equation is to be ${{\boldsymbol{I}}}_{(1123{{\rm{cm}}}^{-1})}$ = 734 × ***C***_*glucose*_ + 5302 (*R* = 0.966). The detection limit of glucose in this case is estimated to be 0.1 × 10^−4^ M using the IUPAC method. It can be seen that, compared to pure Ag NPs, Ag NP@N-GQD exhibit remarkable improvement in SERS performance and show an even lower detection limit and a broader linear range in glucose detection. This result also demonstrated that Ag NP@N-GQD can preserve a large number of hot spots for surface enhancement even after drying.

**Table 1:** Performance comparison of different glucose sensing techniques.

SERS probe materials	Linear range	LOD (Method of determination)	Storage time (days)	Storage condition	Ref
Ag@AuNPs modified GO	2.0–6.0 mM	1.0 mM	30^*^	4 °C	62
Chemical etching of silver nanoparticles	0.01–20 mM.	10 μM	—	—	63
Multi-branched gold nanostructures	5–20 mM	5 mM (IUPAC)	—	—	64
Gold nanoparticles onto 3-MBA/1-DT	2–16 mM	0.5 mM	—	—	52
Ag nanocubes	0–250 mM		—	—	65
Silver nanorod arrays	0–20 mM		—	—	66
silver coating Hollow agarose microneedle	5–150 mM		—	—	60
Pure Ag NP	10^−4^–1 M	10 μM (IUPAC)	30	Normal indoor condition^†^	This work
		0.7 μM (AOAC)			
Ag NP@N-GQD	10^−6^–1 M	0.10 μM (IUPAC)	30	Normal indoor condition^†^	This work
		0.022 μM (AOAC)			

LOD is the acronym of “Limit Of Detection”. The details about the methods of LOD determination are described in the section–“SERS based glucose detection” of the text. ^*^Quantitative information about comparison in SERS performance before and after storage was not available in these reports, although the authors claimed that the SERS substrates worked well after the specified storage period. ^†^Normal indoor condition refers to an average temperature of 25 °C and a relative humidity of 69%.

### Stability in SERS performance after prolonged storage

**Figure 6:**
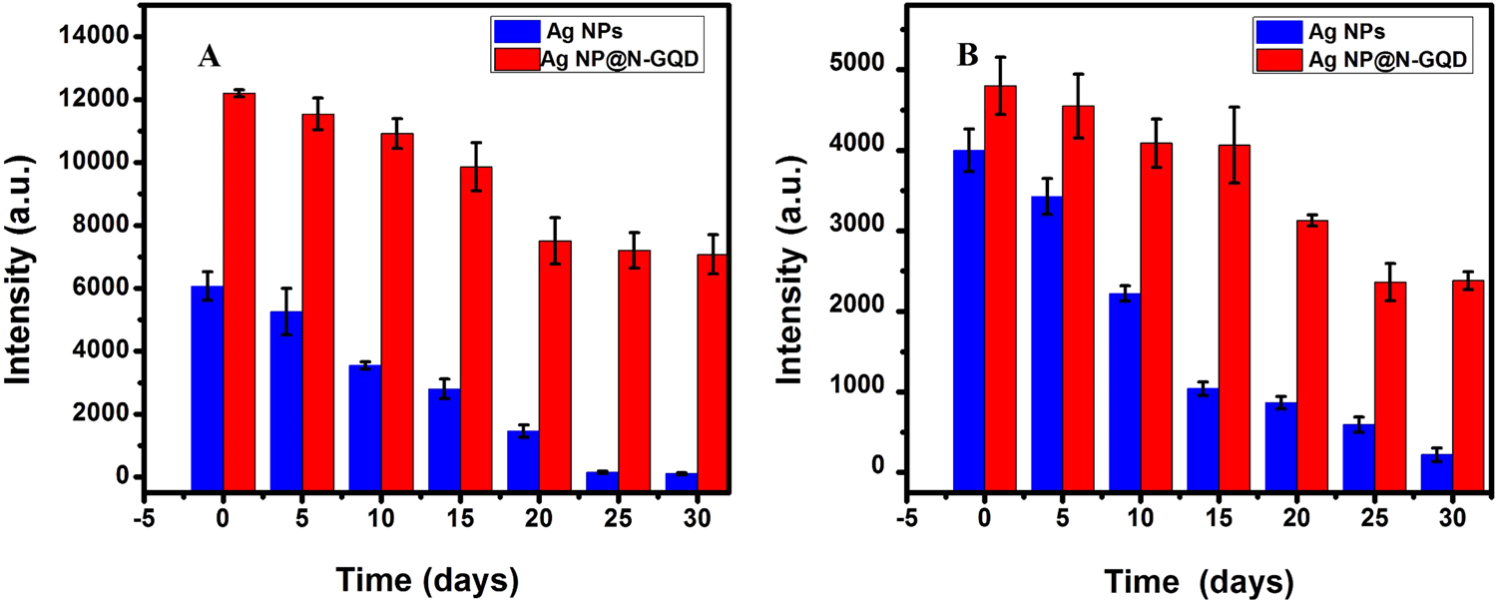
SERS intensity at 1311 cm^−1^ of R6G (1 μM) using Ag NPs (black) and Ag NP@N-GQD (red) after the NPs were stored (**A**) in solution and (**B**) in the dried form, for different numbers of days recorded over 30 days, respectively. Each data point represents the average value from five SERS spectra measured from different locations. Error bars indicate the standard deviations.

Long-term stability is an important parameter for an applicable SERS substrate. We first investigated the stability of the Ag NPs and Ag NP@N-GQD after storage for at least 30 days by measuring the SERS spectra of R6G solution at a concentration of 10^−6^ M. The peak intensity at 1311 cm^−1^ was plotted as a function of storage time as shown in Fig. 6. Two different storage strategies were investigated, including (A) storing NP suspension in a screw-cap centrifuge tube (Tube 50 ml, Greiner Bio-one GmbH, Germany) under normal indoor conditions with an average temperature of 25 °C and a relative humidity of 69% and (B) storing NPs that have been dried on aluminum foil in a normal storage cabinet that was not air proof and also under normal indoor conditions. Note that the volume of NP suspension dried on each piece of the aluminum foil in strategy (B) was identical to that of NP suspension in strategy (A). For stored NP suspension in strategy (A), ultrasonic processing was applied for 30 minutes to disperse nanoparticles prior to use. Then the NP suspension was mixed with R6G solution and sonicated for 5 min before each measurement just as in the measurements using freshly made NPs. While for dried NPs that have been put onto aluminum foil and stored for a period in strategy (B), the R6G solution was directly dropped on the aluminum foil and SERS measurements were performed after 10 min. It can be found from Fig. 6(A) that the SERS intensity measured from the R6G samples containing Ag NP@N-GQD was still 58% of the initial value, i.e. the SERS intensity measured when Ag NP@N-GQD was still fresh, when using Ag NP@N-GQD suspension that have been stored for 30 days after synthesis. Under the same storage condition, the SERS intensity measured from the R6G samples mixed with the Ag NP suspension dropped to about 1.6% after 30 days. We also compared the SERS performance of Ag NP@N-GQD and Ag NPs after drying and then storing for various number of days and a similar trend was found, as shown in Fig. 6(B). After 30 days, the SERS peak intensity values of Ag NP@N-GQD and pure Ag NPs dropped to 50.1% and 5.5% of their initial values, respectively. The results clearly showed that Ag NP@N-GQD can withstand storage for much longer under normal indoor conditions compared to pure Ag NPs. The improved long-term ability can be attributed to the electron-donor effect of the electron-rich N-GQD, in which N-GQD donates its lone pair of electrons to the silver nanoparticle surface and interact with Ag NPs. In addition, the negative charges on the Ag NP@N-GQD could be transferred to the oxidizing agent (O_2_), which can effectively delay the oxidation of Ag NPs in common storage environments^38^.

We also tested the performance of using Ag NP@N-GQD and Ag NPs for SERS based glucose detection after storage for 10 days as shown in Fig. S1(A) and S2(B). The comparison of Figs 5(A,C) and S1 (A,C) showed that the sensitivity, defined as the slope of the line, after 10 days can retain 69.5% of its initial value. In contrast, under the same storage condition, the pure Ag NP SERS data showed that the sensitivity after 10 days only retained 46.9% of its initial value by comparing Figs 5(B,D) and S1 (B,D). These results also prove that Ag NP@N-GQD are more effective in retaining the sensitivity of SERS sensing than pure Ag NPs. Table 1 compares the SERS performance of Ag NP@N-GQD with other earlier SERS based glucose detection methods. It can be seen that the proposed Ag NP@N-GQD exhibits good long-time stability even under normal indoor conditions when compared with other previously reported SERS substrates stored at lower temperature. This result also implies that this novel material is easier to store, carry and have a great potential in the field application.

### SERS detection of glucose in mouse blood samples using Ag NP@N-GQD

The practical efficacy of Ag NP@N-GQD for SERS based glucose detection was evaluated in whole blood samples taken from a BALB/c nude mouse (10 weeks old). The blood was placed in a heparinized tube after being drawn out and then stored at −20 °C, which was thawed for 2 hours at room temperature each time before use. In order to decrease the auto-fluorescence background in a mouse blood sample^53, 54^, the sample was diluted 10 times with deionized water, which then served as the baseline sample. As shown in Table 2, three stock glucose solutions were each mixed with a portion of the original mouse blood sample to achieve three other blood samples with the same dilution factor as the baseline sample but with the additional increase in glucose concentration by 1 mM, 5 mM and 10 mM, respectively. Each of three blood samples of 20 μL was dropped on dried Ag NP@N-GQD thin film and then measured after 10 min.

**Table 2:** Detection of increase in glucose concentration in mouse blood samples.

Sample	Expected increase in glucose concentration (mM)	Estimated increase in glucose concentration (mM)	Mean Percent Error
1	1	0.96 ± 0.06	–4.0%
2	5	4.68 ± 0.36	–6.4%
3	10	9.60 ± 0.53	–4.0%

As shown in Fig. S2(A), the dried Ag NP@N-GQD coated with 1-DT were used as the control group (curve a), Raman peaks at 889, 1073, and 1125 cm^−1^ that appear in the spectra are contributed by the 1-DT layer on the NPs coating. Fourthermore, an obvious SERS peak can be observed at 1123 cm^−1^ from the blood sample (curve b), which is attributed to the glucose. After glucose solution was added, the SERS peak intensity at 1123 cm^−1^ was increased as expected (curves c and e). The linear relation between the SERS intensity at this peak in mouse blood samples and the increase in glucose concentration can be seen in Fig. S2(B). The glucose concentrations in the blood sample were estimated using the calibration curve shown in Fig. 5(C) and the corresponding standard regression equation. The baseline glucose concentration in the original mouse blood sample after dilution by 10 times was estimated to be 2.1 ± 0.4 mM, which agrees with the common range of glucose concentrations in mammalian blood samples^55^. Such results show that Ag NP@N-GQD could be used for the sensitive detection of glucose in real blood samples. The glucose concentrations in the blood sample were estimated using the calibration curve shown in Fig. 5(C) and the corresponding standard regression equation. The baseline glucose concentration in the original mouse blood sample after dilution by 10 times was estimated to be 2.1 ± 0.4 mM, which agrees with the common range of glucose concentrations in mammalian blood samples^55^. Such results show that Ag NP@N-GQD could be used for the sensitive detection of glucose in real blood samples.

### Cost analysis

In order to investigate the potential of this novel nanocomposite for mass production, we made the cost analysis based on information collected in the laboratory synthesis stage as shown in Table 3. The price of chemical agents AgNO_3_ was quoted from Alfa Aesar (UK), and those of citric acid and dicyandiamide were quoted from Sigma-Aldrich (USA). It can be found that the cost of synthesizing N-GQD in the laboratory stage is very low, which is only 0.33% of the overall cost for synthesizing 5 mg Ag NP@N-GQD when using the optimal recipe (Q_N-GQD_/Q_AgNO3_ = 0.08). This clearly indicates that N-GQD are low-cost yet highly effective protective agent. Thus, Ag NP@N-GQD has great potential to be adopted as a cost-effective and sustained SERS substrate for the field test.

**Table 3:** Analysis of itemized cost for synthesizing 5 mg Ag NP@N-GQD, which requires 0.8 mg N-GQD and 10 mg AgNO_3_.

Component	Chemical Agent	ACS number	Pack size	Price (USD)	Chemical Quantity for synthesizing 5 mg Ag NP@N-GQD	Cost (USD)	Cost distribution
Ag NP	AgNO_3_	7761–88–8	5 g	24.9	10.0 mg	4.98 × 10^−2^	99.67%
N-GQD	Citric acid	77-92-9	500 g	90.3	0.8 mg	1.45 × 10^−4^	0.29%
	Dicyandiamid	461-58-5	1000 g	45.7	0.4 mg	1.83 × 10^−5^	0.04%

The measured baseline glucose level (diluted by 10 times) is 0.21 mM. The first and second values in the column of “Estimated increase in glucose concentration” give the mean value and the standard deviation, respectively. Note that the Mean Percent Error is calculated as (Δ_Estimated_ − Δ_Expected_)/Δ_Expected_ × 100%, where Δ_Estimated_ represents the mean estimated increase in glucose concentration and Δ_Expected_ represents the expected increase in glucose concentration.

## Conclusion

We report our attempt towards SERS based field biosensing, which involves the development and characterization of a novel sustained and cost effective substrate composed of silver nanoparticles (Ag NPs) protected by small-sheet nitrogen-doped Graphene Quantum Dots (N-GQD), i.e. Ag NP@N-GQD. The new substrate was synthesized and optimized under mild experimental conditions, which can preserve SERS performance under normal indoor conditions for at least 30 days in both the wet and dry state. Ag NP@N-GQD was demonstrated as a SERS substrate for glucose detection in both aqueous solutions and mouse blood samples. The results suggest that Ag NP@N-GQD nanostructure has great potential to be used as a cost effective and sustained SERS substrate in field biosensing.

## Materials and Methods

### Reagents

Citric acid (CA), dicyandiamide (DCD) and Rhodamine 6 G (R6G) were purchased from Sigma-Aldrich (Saint Louis, Missouri, USA). Silver nitrate (AgNO_3_), D-Glucose anhydrous, sodium hydroxide (NaOH) and 1-decanethiol (1-DT) were ordered from Alfa Aesar (Heysham, Lancashire, UK). Triton X-100 Detergent was purchased from Bio-Rad (Laboratories, Hercules, USA). Hydroxylamine hydrochloride (HH) was ordered from MP Biomedicals (solon, Ohio, USA). Nanopure water (18.3 MΩ · cm) was used throughout the experiments. All reagents were of analytical grade and used as received.

### Instrumentation

A portable Raman spectrometer system (InnoRam-785S, B&W Tek, Newark, Delaware, USA) coupled with a video microscope accessory (BAC151A, B&W Tek, Newark, Delaware USA) was used to measure all spectra. Elmasonic (Elma E30H, Elma, Wetzikon, Switzerland) was used as reaction power and also to disperse nanoparticles. The morphologies of the Ag NPs and Ag NP@N-GQD were investigated using a field emission scanning electronic microscope (FESEM) (JOEL JSM-6700F, JOEL, Tokyo, Japan) system with an accelerating voltage of 5 kV. Transmission electron microscopy (TEM) and High-resolution TEM (HRTEM) measurements were carried out by using a JEOL JEM2100F JOEL, Tokyo, Japan). UV-VIS spectroscopy was carried out using a UV spectrophotometer (UV-2800, UNICO, Dayton, New Jersey, USA). XPS measurements were performed on a Thermo ESCALAB VG Scientific 250 spectrometer equipped with monochromatized Al Ka excitation (ESCALAB 250, Thermo Fisher Scientific, Waltham, Massachusetts, USA).

### Preparation of N-GQD, Pure Ag NPs and Ag NP@N-GQD

According to our previous work^56^, N-GQD were prepared by the hydrothermal treatment of the mixture of CA (2.0 g) and DCD (1.0 g) in 5 mL nanopure water. The mixture was collected after heating at 180 °C for 12 h. After centrifuging treatment at 10000 rpm for 10 min, the product was dispersed in 100 mL nanopure water to obtain the N-GQD stock solution (about 20 mg mL^−1^).

Ag NPs were synthesized through an ultrasonic process at normal temperature^46, 57^. First, 1 mL AgNO_3_ (10 mg mL^−1^) was added into 44 mL Nanopure water (18.3 M · Ω cm). Then 33 μL Triton X-100 was mixed with a total of 5 mL solution with HH (0.03 mM) and NaOH (0.15 mM). After the mixture was added into the above AgNO_3_ solution, the colorless solution turned dark immediately indicating the formation of Ag NPs. Then the resulting solution was sonicated for 30 min. Finally, the Ag NPs were purified by performing 10-min centrifugation at 8000 rpm twice to remove unused agents.

Ag NP@N-GQD were synthesized by adopting the same approach as synthesizing Ag NP^58^. Briefly, 4 mL N-GQD (0.2 mg mL^−1^) was added into 1 mL AgNO_3_ (10 mg mL^−1^) solution before adding 5 mL solution of HH (0.03 mM) and NaOH (0.15 mM) that contained 33 μL Triton X-100. The subsequent steps were identical to those above for synthesizing Ag NPs.

### Raman Measurements

The SERS measurements of R6G solution adsorbed on Ag NPs and Ag NP@N-GQD nanoparticles were conducted using a portable Raman spectrometer system coupled to a microscope accessory as mentioned earlier. The suspension of Ag NPs or Ag NP@N-GQD mixed with R6G solution (V_Ag NPs or Ag NP@N-GQD_/V_R6G_ = 1:1) was each sonicated for 5 min. In every Raman measurement, the solution was dropped into a small ring with a diameter about 5 mm made on aluminum foil for measurements, since aluminum foil yields minimal fluorescence background. The excitation power density was 9.09 W/mm^2^ for all SERS measurements. The Raman spectra of R6G solutions and glucose solutions (and blood samples for glucose detection) were collected with the exposure time values of 500 ms and 5000 ms, respectively. For each sample, Raman measurements were taken from five different locations and then averaged.

### SERS based glucose detection

It is well known that the Raman cross section of glucose molecules is small and its adsorption on the surface of metal nanoparticles is weak, which makes it challenging to detect glucose directly using surface enhanced Raman spectroscopy. According to the previous reports^59, 60^, 1-DT was used to modify the surface of all NPs in the glucose quantification measurements. For this purpose, 1 mL Ag NPs (1 mg mL^−1^) or Ag NP@N-GQD (1 mg mL^−1^) were mixed with 1 mL 1-DT (0.1 mM) overnight. Then 40 µL mixed solution was dropped into the small ring made on aluminum foil and left dry in the air. This layer of 1-DT molecules can capture glucose molecules in close vicinity to Ag nanoparticles to facilitate glucose sensing.

### Analytical enhancement factor (*EF*)

The enhancement factor of AgNP and AgNP@N-GQD (Q_N-GQD_/Q_AgNO3_ = 0.08) for R6G measurements, i.e. *EF*_SERS/Raman_, _R6G_, can be calculated as,

$$EF=\frac{{I}_{1365,SERS}}{{I}_{1365,{\rm{Raman}}}}\times \frac{{P}_{{\rm{Raman}}}}{{P}_{{\rm{SERS}}}}\times \frac{{C}_{{\rm{Raman}}}}{{{\rm{C}}}_{{\rm{SERS}}}}$$

where the two numbers ${I}_{1365,SERS}$ and ${I}_{1365,{\rm{Raman}}}$ are the SERS and ordinary Raman intensities of R6G at 1365 cm^−1^, respectively, at the concentrations of 10^−6 ^M (C_SERS_) and 10^−2^ M (C_Raman_), excited at the corresponding laser power of 50 mW (P_SERS_) and 100 mW (P_Raman_)^46^.

### Detection limit estimation

There are two common methods for the determination of the detection limit. One is to define the detection limit as the glucose concentration yielding the signal intensity equal to three times the standard deviation of the signal intensity measured from a reference solution without any glucose, which was presented by the Association of Analytical Communities (AOAC)^61^. This will be named as the AOAC method in the subsequent text. The other is to identify the smallest glucose concentration at which the signal intensity can be differentiated from that of the reference solution without any glucose by a statistical test, which was presented by the International Union of Pure and Applied Chemistry (IUPAC)^61^. This method will be named as the IUPAC method in the subsequent text. Typically, the former method yields a smaller value for the detection limit. However, the latter method is preferred in this work because we believe that the detection limit obtained this way offers more meaningful practical guidance.

## Electronic supplementary material

Supplementary Information
