# Supplementary material for: Sustained and Cost Effective Silver Substrate for Surface Enhanced Raman Spectroscopy Based Biosensing
Source: Sci Rep. 2017 Jul 31;7:6917. doi: 10.1038/s41598-017-07186-9 (PMC5537298; doi:10.1038/s41598-017-07186-9)
Supplement: Supplementary file 1 — Supplementary Information [file 41598_2017_7186_MOESM1_ESM.doc]

Supplementary Information

**Sustained and Cost Effective Silver Substrate for Surface Enhanced Raman Spectroscopy Based Biosensing**

**Jian Ju ^a^, Wei Liu ^a^, Clint Michael Perlaki ^a^, Keren Chen ^a^, Chunhua Feng ^a,b^ and**

**Quan Liu ^a^,***

**^a^ School of Chemical and Biomedical Engineering, Nanyang Technological University, 70 Nanyang Drive, Singapore 637457, Singapore**

**^b^ School of Environment and Energy, South China University of Technology, Guangzhou 510006, China**

*Correspondence to email: [quanliu@ntu.edu.sg](mailto:quanliu@ntu.edu.sg)

**
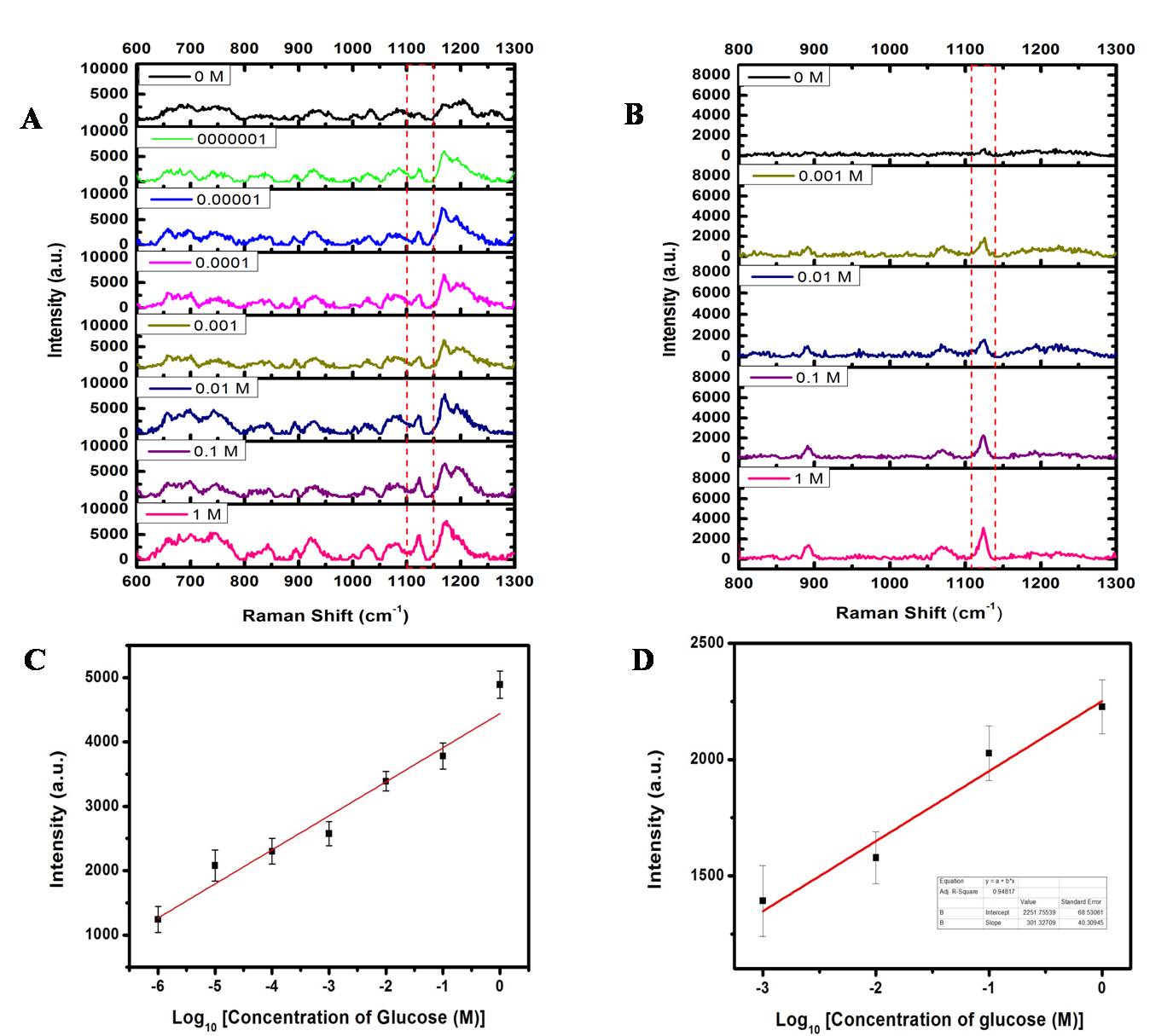
**

Figure S1 Representative SERS spectra of the aqueous glucose solutions mixed with dried 1-DT-modified (A) Ag NP@N-GQD and (B) Ag NPs after the NPs were stored for 10 days. The excitation wavelength was 785 nm with a power density of 9.09 W/mm^2^ and an exposure time of 5000 ms. The SERS intensities for the peaks at 1123 cm^-1^ of the (C) Ag NP@N-GQD and (D) Ag NPs are plotted with respect to the concentration of glucose in the ranges of (10^-6^ to 1 M) and (10^-3^ to 1 M), respectively. Error bars show the standard deviations.


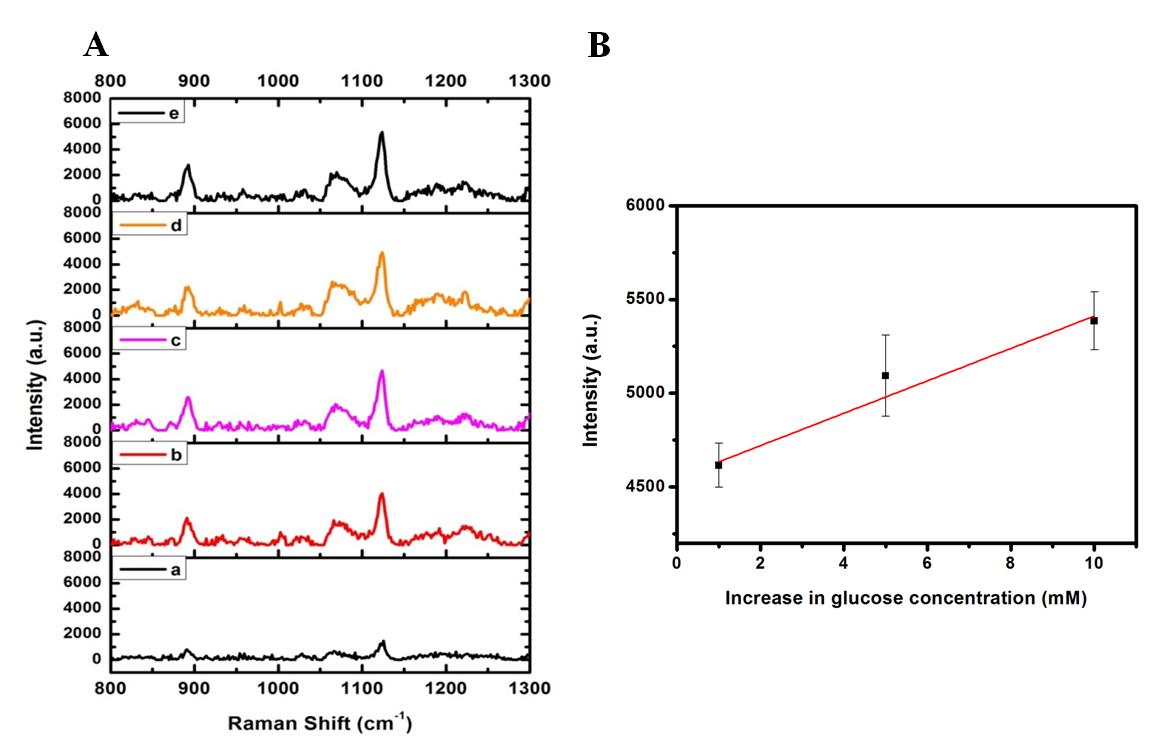


Figure. S2 (A) Representative SERS spectra of control samples, i.e. dried Ag NP@N-GQD (curve a), and mouse　blood samples with different additional glucose concentrations, including the original mouse blood diluted by 10 times with deionizedwater (curve b), the diluted mouse blood samples with additional glucose at a series of concentration of 1 mM (curve c), 5 mM　(curve d) and 10 mM (curve e), respectively. SERS measurements were performed with an excitation wavelength of 785 nm, a power density of 9.09 W/mm^2^and an exposure time of 5000 ms. (B) SERS intensity at 1123 cm^-1^ of mouse blood samples as a function of the increase in glucose concentration from 1mM to 5 mM and then to 10 mM from the baseline value.
